# Parasitism on domestic cats by *Amblyomma auricularium* and serological evidence of exposure to *Rickettsia amblyommatis*

**DOI:** 10.1590/S1984-29612024015

**Published:** 2024-03-18

**Authors:** Ila Ferreira Farias, Glauber Meneses Barboza de Oliveira, Erisson Victor Macedo Lima, Marcelo Bahia Labruna, Mauricio Claudio Horta

**Affiliations:** 1 Laboratório de Doenças Parasitárias, Universidade Federal do Vale do São Francisco - UNIVASF, Petrolina, PE, Brasil; 2 Pós-graduação em Ciências Veterinárias no Semiárido, Universidade Federal do Vale do São Francisco - UNIVASF, Petrolina, PE, Brasil; 3 Laboratório de Doenças Parasitárias, Departamento de Medicina Veterinária Preventiva e Saúde Animal, Faculdade de Medicina Veterinária e Zootecnia, Universidade de São Paulo - USP, São Paulo, SP, Brasil

**Keywords:** Ixodida, Rickettsia, Felis catus domesticus, serology, northeast, Ixodida, Rickettsia, Felis catus domesticus, sorologia, nordeste

## Abstract

The domestic cat is not considered a primary host for any specific tick species; however, it can be affected by some Ixodidae species, such as *Rhipicephalus sanguineus sensu lato* and *Amblyomma* spp. The study reports parasitism by *Amblyomma auricularium* and the detection of anti-*Rickettsia* spp. antibodies in domestic cats from a rural property in the Afrânio municipality, Pernambuco, Brazil. *Amblyomma auricularium* (24 nymphs, six females, and four males) and *Amblyomma* sp. (42 larvae) parasitized three cats, and 73 free-living ticks were captured in armadillo burrows: *A. auricularium* (36 nymphs, six females, five males) and *Amblyomma* sp. (26 larvae). Blood samples from cats were collected and the obtained plasma were subjected to indirect immunofluorescence assay (IFA) to detect antibodies against *Rickettsia* antigens. Thus, anti-*Rickettsia* spp. antibodies were determined (titers ranging from 128 to 512) and showed a predominant antibody response to *Rickettsia amblyommatis* or a very closely related genotype. This study reports the first infestation of nymphs and adults of *A. auricularium* on cats in a new area of occurrence in the semi-arid region of Northeastern Brazil and reports for the first time the presence of anti-*Ricketsia* antibodies in cats in the region, with *R. amblyommatis* as the probable infectious agent.

## Resumo

O gato doméstico não é considerado hospedeiro para nenhuma espécie de carrapato. Pode, entretanto, ser afetado por algumas espécies de Ixodídeos, como *Rhipicephalus sanguineus* e *Amblyomma* spp. Este estudo relata o parasitismo por *Amblyomma auricularium* e detecção de anticorpos anti-*Rickettsia* spp. em gatos domésticos, em uma propriedade rural no município de Afrânio, Pernambuco, Brasil. *Amblyomma auricularium* (24 ninfas, seis fêmeas e quatro machos) e *Amblyomma* sp. (42 larvas) parasitavam os gatos, e 73 carrapatos de vida-livre foram capturados em tocas de tatus: *A. auricularium* (36 ninfas, seis fêmeas, cindo machos) e *Amblyomma* sp. (26 larvas). Amostras de sangue dos três gatos foram coletadas, e os plasmas obtidos foram submetidos à reação de imunofluorescência indireta (RIFI) para a detecção de anticorpos contra antígenos de *Rickettsia*. Dessa forma, anticorpos anti-*Rickettsia* spp. foram determinados (títulos variando de 256 a 512) e demonstraram uma resposta predominante para *Rickettsia amblyommatis* ou genótipo próximo. Este estudo relata, pela primeira vez, infestação de ninfas e adultos *A. auricularium* em gatos em uma nova área de ocorrência na região semiárida do nordeste do Brasil. E, também relata, pela primeira vez, a presença de anticorpos anti-*Ricketsia* em gatos na região, com *R. amblyommatis* como agente provável pela infecção.

Domestic cats (*Felis catus domesticus*) are parasitized by a variety of ectoparasites including mites, lice, fleas, and ticks (Dantas-Torres & Otranto, 2014), which are of great importance in feline medicine (Ferreira et al., 2010). Felines are reservoirs for various pathogens with zoonotic potential for humans, including *Bartonella henselae*, which causes cat scratch disease and *Rickettsia typhi*, which causes murine typhus (Case et al., 2006), both of which are transmitted by fleas.

Ticks are of great importance to human and animal health as they are vectors of pathogens, and many species feed on a wide range of vertebrate hosts, including cats (Mendes-de-Almeida et al., 2011). The domestic cat is not considered the primary host of any tick species, however, ectoparasitism has already been reported by some ixodids, including *Rhipicephalus sanguineus* sensu lato (s.l.) (Horta et al., 2007; Ferreira et al., 2010; Mendes-de-Almeida et al., 2011), *Amblyomma triste* (Silva et al., 2007), *Amblyomma sculptum*, *Amblyomma aureolatum,* and *Amblyomma ovale* (Horta et al., 2007; Dantas-Torres & Otranto, 2014).

The genus *Amblyomma* has the greatest species diversity in Brazil, and its distribution has been reported worldwide (Barros-Battesti et al., 2006). Rickettsiae are a group of obligate intracellular Gram-negative bacteria that cause a variety of diseases called rickettsioses, transmitted by hematophagous arthropods (Hiraoka et al., 2005). There have been reports of hemoparasitoses in cats caused by *Ehrlichia canis* (Oliveira et al., 2009; André et al., 2017), *Anaplasma* spp. (André et al., 2014, 2017), *Mycoplasma* spp. (André et al., 2017; Pesqueira et al., 2023), *Babesia* spp., *Hepatozoon* spp. (André et al., 2015), *Theileria* spp. (André et al., 2014, 2015), *Bartonella* spp. (André et al., 2014), and *Cytauxzoon* (André et al., 2017). However, few studies in Brazil have described *Amblyomma* spp. infestations and the determination of anti-*Rickettsia* antibodies (Horta et al., 2007).

Thus, this study reports the parasitism by *Amblyomma auricularium* in three domestic cats from a farm in the municipality of Afrânio, state of Pernambuco, Brazil, in addition to the detection of anti-*Rickettsia* spp. antibodies in plasma samples.

From September to December 2021, 76 specimens of ticks were collected from the three domestic cats that used to live on a farm in the municipality of Afrânio. In addition, 73 tick specimens were collected from inside six burrows previously inhabited by six-banded armadillos (*Euphractus sexcinctus*) on the same farm. To collect ticks from the burrows, we used a flannel with a rod inserted into the burrows, and when the flannel was removed, the ticks were visualized on it. All the ticks were placed in tubes containing 70% ethanol and transported to the laboratory for taxonomic identification. Ticks were separated by stages and identified according to Barros-Battesti et al. (2006) and Martins et al. (2010).

Blood samples were collected through venipuncture of the cephalic vein, previously disinfected with ethanol 70%, using 0.55 × 20 mm needles and sterile syringes. Samples were stored in sterile tubes containing anticoagulants at room temperature. Blood samples were centrifuged at 5,000 rpm for 15 min to obtain plasma, which was stored at -20ºC until laboratory analysis. Plasma samples from cats were individually tested by IFA to verify their reactivity to *Rickettsia* spp., according to Horta et al. (2007), using crude antigens of *Rickettsia rickettsii* strain Taiaçu, *Rickettsia parkeri* strain At24, *Rickettsia amblyommatis* strain Ac37, *Rickettsia rhipicephali* strain HJ5, and *Rickettsia bellii* strain Mogi. Each *Rickettsia* strain was cultivated in Vero cells and harvested when nearly 100% of the cells were infected. The infected cells were centrifuged at 4,000 g for 10 min, and pellet was washed in 0.1 M phosphate-buffered saline (PBS), pH 7.4, centrifuged again, and resuspended in PBS containing 1% bovine calf serum and 0.1% sodium azide. Ten microliters of rickettsiae-infected cells were applied onto each of 12 wells on microscopic slides, air-dried, fixed in acetone for 10 min, and stored at -80ºC until used. Feline plasma was diluted in two-fold increments with PBS starting from a 1:64 dilution. Ten microliters of diluted plasma were added to each well of the antigen slides. The slides were incubated at 37ºC for 30 min in a humid chamber. The slides were rinsed once, and then washed twice for 15 min per wash in PBS. The slides were incubated with fluorescein isothiocyanate-labeled goat anti-cat IgG (Sigma, St Louis, USA) from a 1:1,000 dilution, and washed as described earlier. The slides were mounted with buffered glycerin under coverslips. The slides were read using an ultraviolet microscope (Olympus, Tokyo, Japan) at 400x magnification. Plasma was considered to contain antibodies against the rickettsiae if it displayed a reaction at the 1:64 dilution. End-point titers against each *Rickettsia* strain were determined by testing serial plasma dilutions. In each slide, a serum previously shown to be non-reactive (negative control) and a known reactive cat serum (positive control) were tested (Horta et al., 2007).

The ticks collected from cats were identified as *A. auricularium* (24 nymphs, six females, four males) and *Amblyomma* sp. (42 larvae). The ticks collected from the armadillo burrows were identified as *A. auricularium* (36 nymphs, six females, and five males) and *Amblyomma* sp. (26 larvae). The presence of anti-*Rickettsia* spp. antibodies was detected in all cats: cat#1 reacted to *R. amblyommatis* (titer 512) and *R. rhipicephali* (128); cat#2 reacted to *R. rickettsii* (128), *R. amblyommatis* (512), and *R. rhipicephali* (256); and cat#3 reacted to *R. amblyommatis* (titer 512) and *R. rhipicephali* (256). Cat#1 showed antibody titers for *R. amblyommatis* that were at least four-fold higher than those for the other three rickettsial antigens (titer 512), indicating a predominant antibody response to *R. amblyommatis* or a very closely related genotype.

This study assessed one male and two female healthy adult felines. The three cats had free access to a wooded area where wild animals such as rodents, marsupials, and armadillos were present, and it was possible to visualize and count six empty armadillo burrows near the property. The coexistence of domestic animals with wildlife in forest areas can favor tick infestation, thus facilitating the transmission of pathogens from wild animals to domestic animals and humans (Little et al., 2018).

The *A. auricularium* tick is a parasite of armadillos (Chlamyphoridae and Dasypodidae), which occur in the Neotropical and Nearctic regions (Guglielmone et al., 2003). However, despite its moderate specificity for armadillo species such as *Dasypus novemcinctus* Linnaeus 1758 (Guglielmone et al., 2003), there are reports of parasitism on other wild animals: *Conepatus semistriatus* Boddaert, 1785 (Saraiva et al., 2013), *Tamandua tetradactyla* Linnaeus, 1758 (Costa et al., 2020), *Thrichomys apereoides* Lund, 1839, *Monodelphis domestica* Wagner, 1842, *Galea spixii* Wagler, 1831 (Horta et al., 2011), *Nasua narica* Linnaeus, 1766 (Bermúdez et al., 2021), *Thrichomys laurentius* Thomas, 1904 (Oliveira et al., 2020), *Thrichomys inermi*s Pictet, *Monodelphis domestica* Wagner, 1842, (Maia et al., 2018) *Lycalopex vetulus* Lund 1842, *Dicotyles tajacu* Lineu, 1758 (Martins et al., 2020) various bird species (Lugarini et al., 2015). In domestic animals, parasitism has been reported on cattle, dogs, and horses (Guglielmone et al., 2003). In the present study, we report for the first time the infestation of *A. auricularium* on domestic cats.

Studies reporting tick parasitism and serological evidence of exposure to *Rickettsia* spp. in cats are very rare in Brazil because cats are not the primary hosts of any tick species and thus far have no importance in the epidemiological cycle of Rickettsiae. A study carried out by da Silva et al. (2007) in the state of Rio Grande do Sul reported parasitism of a cat by *Amblyomma triste*, while Mendes et al. (2019) reported parasitism by *Amblyomma sculptum* in the state of Rio de Janeiro; however, no diagnosis was made to prove exposure to agents from the Rickettsiae group.

In a previous study conducted in the same region as the present study, Saraiva et al. (2013) demonstrated the infection and vectorial competence of *A. auricularium* to *R. amblyommatis*. In this study, we confirmed, for the first time, the exposure of cats to the spotted fever group Rickettsiae, likely *R. amblyommatis*, using IFA. Generally, studies that detect anti-*Rickettsia* antibodies included included dogs, horses, ruminants, or wild animals, and those dealing with domestic cats in Brazil are scarce. Studies from other countries have reported Rickettsiae in cats and ticks, highlighting their epidemiological importance (Hiraoka et al., 2005; Case et al., 2006; Segura et al., 2014; Little et al., 2018).

Lopes et al. (2018) and Oliveira et al. (2020) reported the presence of anti-*R. amblyommatis* antibodies in *Didelphis albiventris* and *Rattus rattus*. Here, we found anti- *R. amblyommatis* antibodies in domestic cats, thus suggesting the likely circulation of *R. amblyommatis* in both wild and domestic animals. *R. amblyommatis*, initially considered a non-pathogenic bacterium for humans, has already been considered a possible human pathogen based on serological evidence of human infection in the United States (Apperson et al., 2008).

In Brazil, there are at least two distinct reports of *A. auricularium* parasitism on humans (Lopes et al., 2018; Szabó et al., 2020). As a result, the true zoonotic potential of these findings remains unclear. However, it underscores the importance of implementing epidemiological surveillance to monitor the prevalence of this spotted fever group Rickettsiae and its vector in the region, using felines with free access to forest areas and hunting habits as sentinels for this pathogen.

The study reports for the first time the infestation of nymphs and adults of *A. auricularium* on domestic cats in a new area of occurrence in the semi-arid region of Northeast. Additionally, the study also focused on the detection of anti-*Rickettsia* spp. antibodies in parasitized cats, with *R. amblyommatis* identified as the probable infectious agent.
